# The coagulation-related genes for prognosis and tumor microenvironment in pancreatic ductal adenocarcinoma

**DOI:** 10.1186/s12885-023-11032-9

**Published:** 2023-06-29

**Authors:** Di Wang, Song-ping Cui, Qing Chen, Zhang-yong Ren, Shao-cheng Lyu, Xin Zhao, Ren Lang

**Affiliations:** Department of Hepatobiliary and Pancreaticosplenic Surgery, Beijing Chaoyang Hospital, Capital Medical University, No. 8 Gongtinan Road, Chaoyang District, Beijing, 100020 People’s Republic of China

**Keywords:** Pancreatic ductal adenocarcinoma, Coagulation, Tumor microenvironment, Stratified model, ITGA2

## Abstract

**Background:**

Pancreatic ductal adenocarcinoma (PDAC) is a malignancy characterized by challenging early diagnosis and poor prognosis. It is believed that coagulation has an impact on the tumor microenvironment of PDAC. The aim of this study is to further distinguish coagulation-related genes and investigate immune infiltration in PDAC.

**Methods:**

We gathered two subtypes of coagulation-related genes from the KEGG database, and acquired transcriptome sequencing data and clinical information on PDAC from The Cancer Genome Atlas (TCGA) database. Using an unsupervised clustering method, we categorized patients into distinct clusters. We investigated the mutation frequency to explore genomic features and performed enrichment analysis, utilizing Gene Ontology (GO) and Kyoto Encyclopedia of Genes (KEGG) to explore pathways. CIBERSORT was used to analyze the relationship between tumor immune infiltration and the two clusters. A prognostic model was created for risk stratification, and a nomogram was established to assist in determining the risk score. The response to immunotherapy was assessed using the IMvigor210 cohort. Finally, PDAC patients were recruited, and experimental samples were collected to validate the infiltration of neutrophils using immunohistochemistry. In addition, and identify the ITGA2 expression and function were identified by analyzing single cell sequencing data.

**Results:**

Two coagulation-related clusters were established based on the coagulation pathways present in PDAC patients. Functional enrichment analysis revealed different pathways in the two clusters. Approximately 49.4% of PDAC patients experienced DNA mutation in coagulation-related genes. Patients in the two clusters displayed significant differences in terms of immune cell infiltration, immune checkpoint, tumor microenvironment and TMB. We developed a 4-gene prognostic stratified model through LASSO analysis. Based on the risk score, the nomogram can accurately predict the prognosis in PDAC patients. We identified ITGA2 as a hub gene, which linked to poor overall survival (OS) and short disease-free survival (DFS). Single-cell sequencing analysis demonstrated that ITGA2 was expressed by ductal cells in PDAC.

**Conclusions:**

Our study demonstrated the correlation between coagulation-related genes and the tumor immune microenvironment. The stratified model can predict the prognosis and calculate the benefits of drug therapy, thus providing the recommendations for clinical personalized treatment.

**Supplementary Information:**

The online version contains supplementary material available at 10.1186/s12885-023-11032-9.

## Introduction

The mortality rate of pancreatic cancer is increasing and is a major contributor to tumor-related deaths due to its poor prognosis [1]. Pancreatic ductal adenocarcinoma (PDAC) is the most common histological type of pancreatic cancer, accounting for over 90% of cases [2]. PDAC is often diagnosed at an advanced stage, making it a significant challenge for surgical therapy [3]. The estimated postoperative 5-year overall survival (OS) rates range between 5 and 10% [4]. Patients with malignant tumors are at an increased risk of both venous thromboembolism (VTE) and arterial thromboembolism [5–7]. The prevalence of VTE varies among different types of tumors, with PDAC and brain tumors having the highest rates [8, 9]. Thus, there may be specific pathways that increase the risk of VTE in these cases. However, the mechanism of thromboembolism in malignant tumor patients is complex and not fully understood. It is generally recognized that the destruction of the vascular wall can result in hemorrhage and intravascular thrombosis, which can also lead to extravascular thrombosis due to increased vascular permeability and intravascular substance overflow [10, 11]. In addition to the previously mentioned mechanisms, other biological processes, such as extracellular vesicle activity, activation of inflammatory cells and formation of neutrophil extracellular traps also play a crucial role in cancer-related VTE in PDAC [12–14].

Emerging evidence suggests that coagulation is closely linked to the tumor microenvironment (TME) [15]. The TME is composed of various cell types, including malignant cells, stromal cells and immune cells, which interact with each other and contribute to tumor growth and migration [16]. A study by Saidak Z demonstrated that the coagulome interacts with the TME and that fibrinolysis is useful in assessing the immune checkpoints or the immune status within the TME [17]. An increasing body of evidence suggests that microscopic intravascular thrombosis in glioblastoma can lead to remodeling of the TME, including the recruitment of immunosuppressive cells, microvascular hyperproliferation and cancer cell migration [18]. Burzynski LC reported that thrombin could activate IL-1α, and pro-IL-1α on macrophages and platelets were also cleaved and activated by thrombin contributing to the inflammatory response [19]. Additionally, anticoagulants exhibit promising potential for enhancing the effectiveness of adjuvant therapy. Ruf W's research indicated that antithrombotic therapy with employing oral FXa inhibitors could mitigate tumor immune evasion and demonstrated the clinical advantages of immunotherapy through targeting innate immune cells [20]. These findings imply that coagulation plays a crucial role in the tumor microenvironment, influencing tumor progression and the inhibition of the immune response.

The advancements in sequencing technology and bioinformatics have brought about a new era in the realm of medical research, allowing us to investigate the correlation between coagulation and TME in a more concrete and tangible way. Through the application of unsupervised clustering algorithms, our study succeeded in identifying various subtypes of genes related to coagulation. We then proceeded to conduct a comparative analysis between two distinct gene clusters, assessing their differences in relation to TME and immunotherapy response. Finally, a comprehensive prognostic model was developed, which encompassed four pivotal genes that were screened using univariate Cox regression analysis, as well as difference and LASSO analysis. Our study delved deeper into the realms of predictive ability, immune infiltration, tumor mutation burden (TMB) and drug therapy benefits across various risk groups, with the aim of providing tailored and personalized recommendations for clinical treatment. Through our rigorous analysis, we successfully identified the hub gene ITGA2, which displayed a high expression level and was linked to poor prognosis in PDAC. Further analysis, conducted via Single cell sequencing techniques, revealed that ITGA2 was primarily expressed by epithelial cells and was intricately involved in the metastatic process of PDAC tumors.

## Materials and methods

### Ethics statements

The study was conducted in accordance with the Declaration of Helsinki (as revised in 2013) and was approved by the Ethics Committee of Beijing Chaoyang Hospital (No. 2020-D-301).

### Data collecting

The coagulation pathways under investigation were derived from the KEGG database (https://www.genome.jp/kegg/), encompassing both Platelet activation and complement and coagulation cascades [21]. Following an extensive screening process, a total of 203 genes associated with these pathways were identified and subsequently classified as coagulation-related genes (Supplementary Table 1). The transcriptome data and clinical records of 178 patients diagnosed with PDAC were collated from the TCGA database, with additional mutation and somatic copy number alteration data obtained from the same source. For further analysis, alteration data and clinical information were sourced from the cbioportal tool [22]. In order to supplement our findings, we also examined the IMvigor210 dataset, which comprised transcriptome data, clinical information and immunotherapy response data for 348 patients diagnosed with urothelial cancer, sourced from the IMvigor210CoreBiologies R package [23].

### Coagulation-related genes molecular patterns

Our study utilized consensus clustering analysis, conducted with the aid of the “ConsensusClusterPlus” R package, to investigate the molecular subtypes present in PDAC [24]. In order to enhance the stability of our results, this analysis was performed a total of 1000 times. Following this, we proceeded to examine the distributional differences present in coagulation clusters via principal component analysis (PCA). Additionally, we conducted a correlation analysis between coagulation clusters and a variety of clinicopathological factors, including age, gender, T, N and histologic grade [25].

### Functional enrichment analyses

Our study utilized Gene Set Enrichment Analysis (GSEA) analysis was conducted to explore the underlying biological pathways present in the data. This analysis was conducted using GSEA software [26]. Additionally, we performed biological process and pathway enrichment analyses through the use of the R clusterProfiler package, with Kyoto Encyclopedia of Genes and Genomes (KEGG) and Gene Ontology (GO) analyses were performed by R clusterProfiler package. ClueGO can visualize and analysis the genes function in the different risk groups [27].

### Tumor microenvironment and immune infiltration

The evaluation of the tumor immune microenvironment was conducted through single-sample Gene Set Enrichment Analysis (ssGSEA) using the “GSVA” algorithm implemented in R software [28]. The infiltration of 16 immunocytes and 13 immune-related functions was analyzed across distinct coagulation clusters. The patients’ immune score, Stromal score, ESTIMATE score and Tumor purity were assessed utilizing the “estimate” R package and ESTIMATE algorithm. The ESTIMATE algorithm utilizes transcriptome sequencing data to estimate the presence of tumor cells and other cell types in the analyzed sample [29].

### Analysis of immune checkpoint and drug sensitivity

We assessed the expression levels of immune checkpoint gene between two groups segregated by risk score. To evaluate the efficacy of ICB, we employed the TIDE score, which was calculated using the TIDE online tool [30]. We also measured the TMB in terms of the number of mutations per million bases in the tumor tissue, which was estimated using the ‘TMEscore’ R packages to determine the response to ICB [31]. Additionally, we explored the relationship between the prognostic genes and chemotherapy drugs using the Drug Gene Interaction Database (DGIdb). The 3D structures of drugs were visualized using the PubChem website [32].

### The construction of the prognostic model

The present study employed LASSO analysis through the “glmnet” package in R software via 1000-folds cross-validation. Clinical parameters were incorporated to examine their correlation with survival outcomes. Our results revealed that Age, LNM and risk score were identified as independent prognostic factors through univariable and a multivariable Cox regression analysis. To construct the nomogram, we utilized the “rms” R package. We assessed the predictive ability of the nomogram through calibration plots and ROC curve [33].

### Single cell sequencing analysis and cell–cell communication

The single-cell transcriptome file of PDAC samples in GSE205013 was acquired from the Gene expression omnibus (GEO) database (http://www.ncbi.nlm.nih.gov/geo/). We selected PDAC specimens obtained through surgical resection of primary pancreatic lesions (*n* = 10) and isolated cells via centrifugation, which were subsequently loaded on a chromium controller (10X Genomics) for analysis [34]. Cells were subsequently filtered based on high-quality parameters, including detection of > 500 genes, > 1500 unique molecular identifiers, and < 15% of transcripts coming from mitochondrial genes. Log-normalization was utilized to normalize the data and the FindVariableFeatures function was employed to identify highly variable genes. Additionally, all genes were scaled using the ScaleData function, followed by principal component analysis (PCA) downscaling. The cells were subsequently clustered using the FindNeighbors and FindClusters, with a set resolution of 0.8, to obtain cell subgroups [35]. The cells were annotated using an annotation approach. To explore the communicating interactions between cells and identify the mechanism of the communicating molecules at the single-cell resolution, we utilized the R package “CellChat” (version 1.0.0) to analyze cells involved in nine cell groups [36].

### Immunochemistry

The present study employed immunohistochemical techniques to evaluate ITGA2 expression in PDAC tissue sections. Prior to analysis, tissue samples underwent a series of preparatory steps including dewaxing, hydration, and immersion in methanol containing 0.3% hydrogen peroxide. Following washing with PBS, tissue samples were blocked using 1% blocking serum for 30 min, and subsequently incubated overnight with primary antibodies against ITGA2 that had been diluted 500 times using antibody dilution. The slides were washed with PBS thrice and subsequently incubated with biotinylated sheep anti-rat IgG for 15 min, followed by three additional PBS washes. The peroxidase reaction was visualized using DAB for 2 min.

## Results

### Identification of coagulation molecular patterns in PDAC

**Fig. 1:**
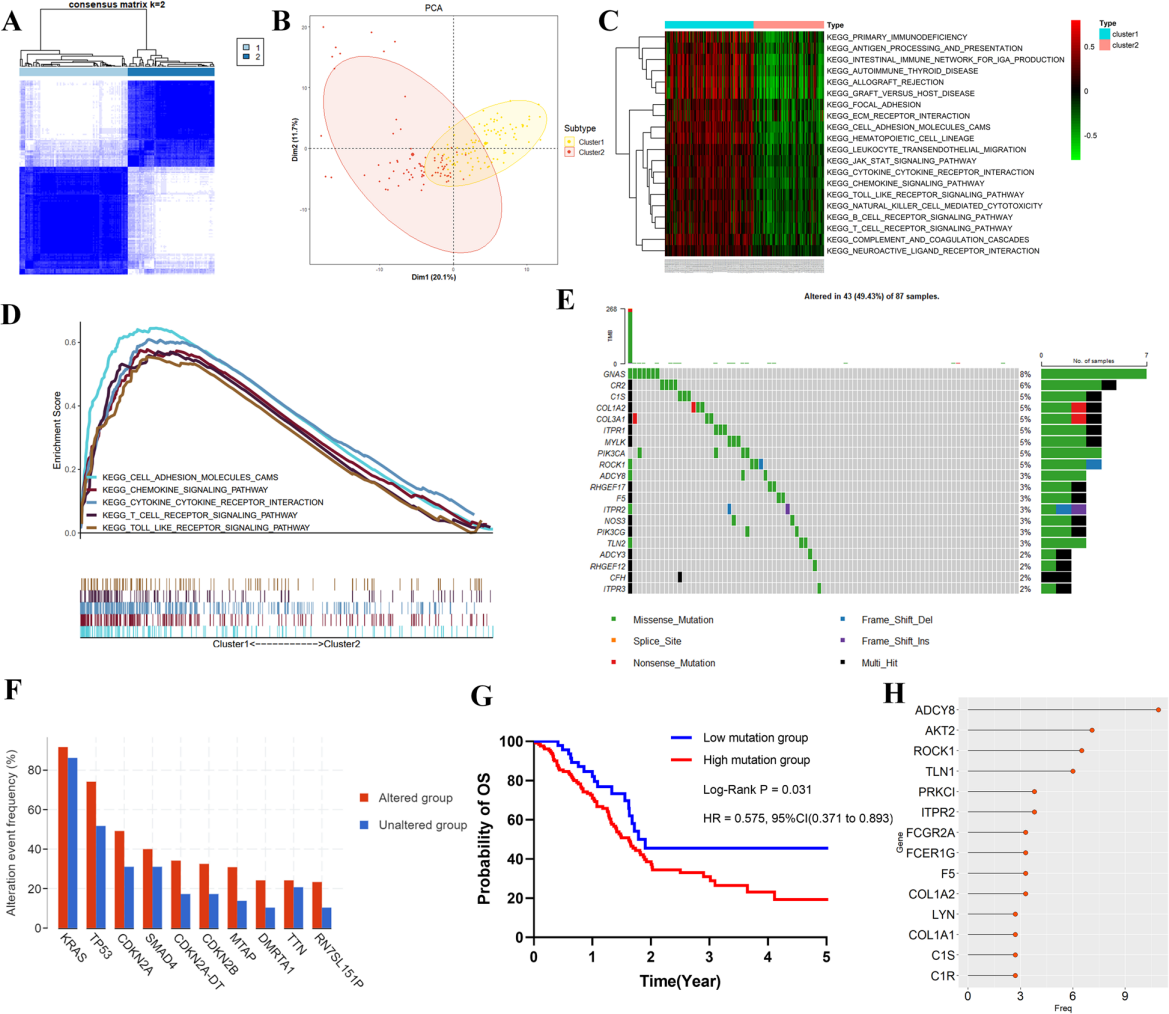
Consensus clustering analysis and enrichment analysis and alterations analysis in PDAC. **A** Two clusters were identified in the TCGA cohort. **B** PCA analysis in two clusters. **C** Heatmap of the KEGG pathways between two clusters. **D** GSEA of 5 enriched pathways in PDAC patients with cluster1. **E** The waterfall graphs of mutated coagulation-related genes. **F** The proportion different genes in altered group and unaltered group. **G** K-M survival analysis of the different mutation group. **H** Lollipop chart of the CNA proportion in coagulation-related genes

We present a novel unsupervised clustering method based on the expression of coagulation-related genes, which identified two distinct coagulation subtypes in 178 PDAC patients. Our analysis revealed that cluster 1 (99, 55.6%) and cluster 2 (79, 44.4%) (Fig. 1A) exhibit significant differences in their transcriptomic profiles, as demonstrated by PCA (Fig. 1B) and a heatmap illustrating the correlation two coagulation patterns and clinicopathological factors (Supplementary Fig. 1A). Moreover, we conducted enrichment analysis and found that cluster 1 was enriched in tumor and inflammatory pathways, including the Focal adhesion pathway, toll-like receptor signaling pathway, T-cell receptor signaling pathway, and B-cell receptor signaling (Fig. 1C). Furthermore, GSEA confirmed significant differences in the toll-like receptor signaling pathway, T-cell receptor signaling pathway and chemokine signaling pathway (Fig. 1D). Our results provide novel insights into the coagulation subtypes in PDAC patients and offer potential targets for therapeutic intervention.

### Mutation analysis of coagulation-related genes

We report here the results of mutation analysis aimed at exploring genomic characteristic of coagulation-related genes in PDAC. Our findings, presented in Fig. 1E, show that the mutation rate in PDAC patients was 49.4%, with a ranged of 2–8% for coagulation-related genes. Among these genes, GNAS had the highest mutation frequency (8%), followed by CR2 (6%), C1S (5%), COL1A2 (5%) and COL3A1 (5%), with Missense_Mutation and Multi_Hit being the main mutation patterns. We observed that patients with altered group had a higher alteration frequency rate of KRAS, TP53 and CDKN2A than unaltered group, as shown in Fig. 1F. Supplementary Fig. 1B indicated that alteration and frequency were categorized into mutation (22.2%), amplification (21.5%), deep deletion (4.0%) and multiple alterations (32.9%), respectively. Furthermore, we sorted the number of mutations for each patient and obtained the optimal cutoff value to divide patients into low and high mutation groups. Ultimately, we identified 50 patients in the low mutation group and 134 patients in the high mutation group for survival analysis. Kaplan–Meier curve suggested patients with a high mutation had a poor prognosis compared with the low mutation group (Fig. 1G). Finally, ADCY8, AKT2, ROCK1 and TLN1 exhibited higher alterations frequency (6.0–10.9%) due to the amplification of coagulation-related genes (Fig. 1H).

### Immune infiltration and tumor microenvironment

**Fig. 2:**
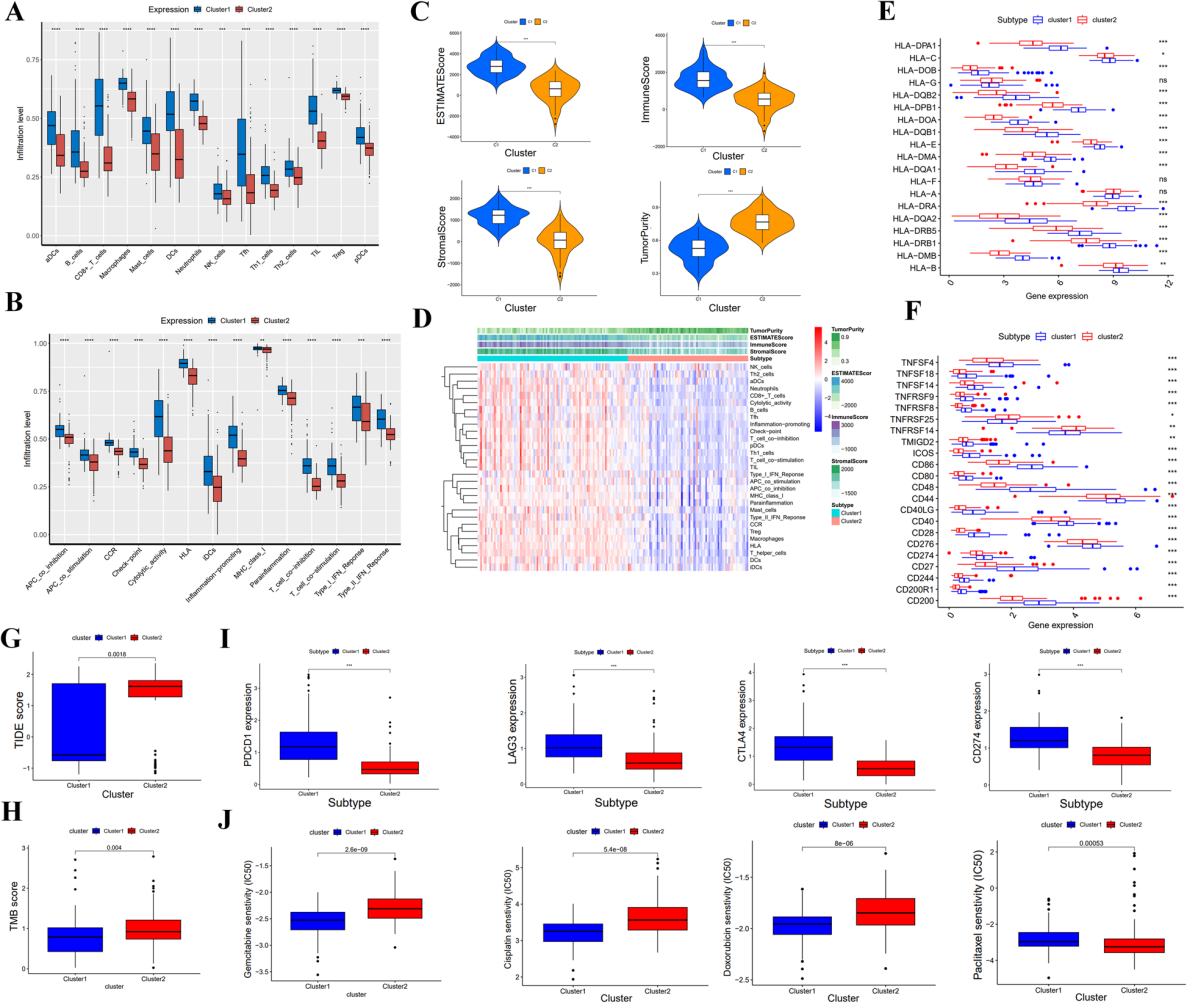
the analysis of tumor immune microenvironment analysis and estimation in immunotherapy and chemotherapy response between two clusters. **A** Comparison of immune cell types and (**B**) immune-related functions. **C** Stromal score, Immune score, ESTIMATE score, Tumor purity in tumor immune microenvironment. **D** Heatmap of the immune cells between two clusters. **E** Gene expression of HLA in two clusters. **F** Gene expression of MHC between two clusters. V The TIDE (**G**) and TMB (**H**) between two clusters. **I** The level of immune checkpoint genes in two clusters. **J** The response of two clusters in four chemotherapy drugs

The present study employs ssGSEA algorithm to investigate the tumor immune microenvironment in two distinct clusters. Cluster 1 showed higher levels of infiltration of various immunocytes, such as Neutrophils, Macrophage and B-cells (Fig. 2A), with a higher proportion of inflammation promoting cells compared to cluster 2 (Fig. 2B). The study further reveals that the Immune score, Stromal score, ESTIMATE score of cluster 1 were significantly higher, whereas Tumor purity was lower (Fig. 2C). We also compared tumor immune microenvironment in two clusters according to ssGSEA algorithm (Fig. 2D). In addition, HLA and MHC proportions were higher in cluster 1 than in cluster 2 (Fig. 2E-F). The study also examined the association between clusters and immunotherapy response by analyzing TMB, TIDE and immune checkpoint genes expression. TIDE score and TMB score were higher in cluster 2 compared to cluster 1 (Fig. 2G-H), while PD-1, PD-L1, CTLA4 and LAG3 expressions were higher in cluster 1 than cluster 2 (*P* < 0.05) (Fig. 2I). Furthermore, the IC50 values for common drugs, such as Gemcitabine, Cisplatin and Doxorubicin were calculated to evaluate drug response in both clusters. IC50 of Gemcitabine, Cisplatin, and Doxorubicin was higher in cluster 2 than in cluster 1, whereas IC50 of Paclitaxel was higher in cluster 1 than in cluster 2 (Fig. 2J). These findings suggest that cluster 1 may respond better to immunotherapy, whereas cluster 2 may benefit from chemotherapy treatment.

### Screening the hub genes

**Fig. 3:**
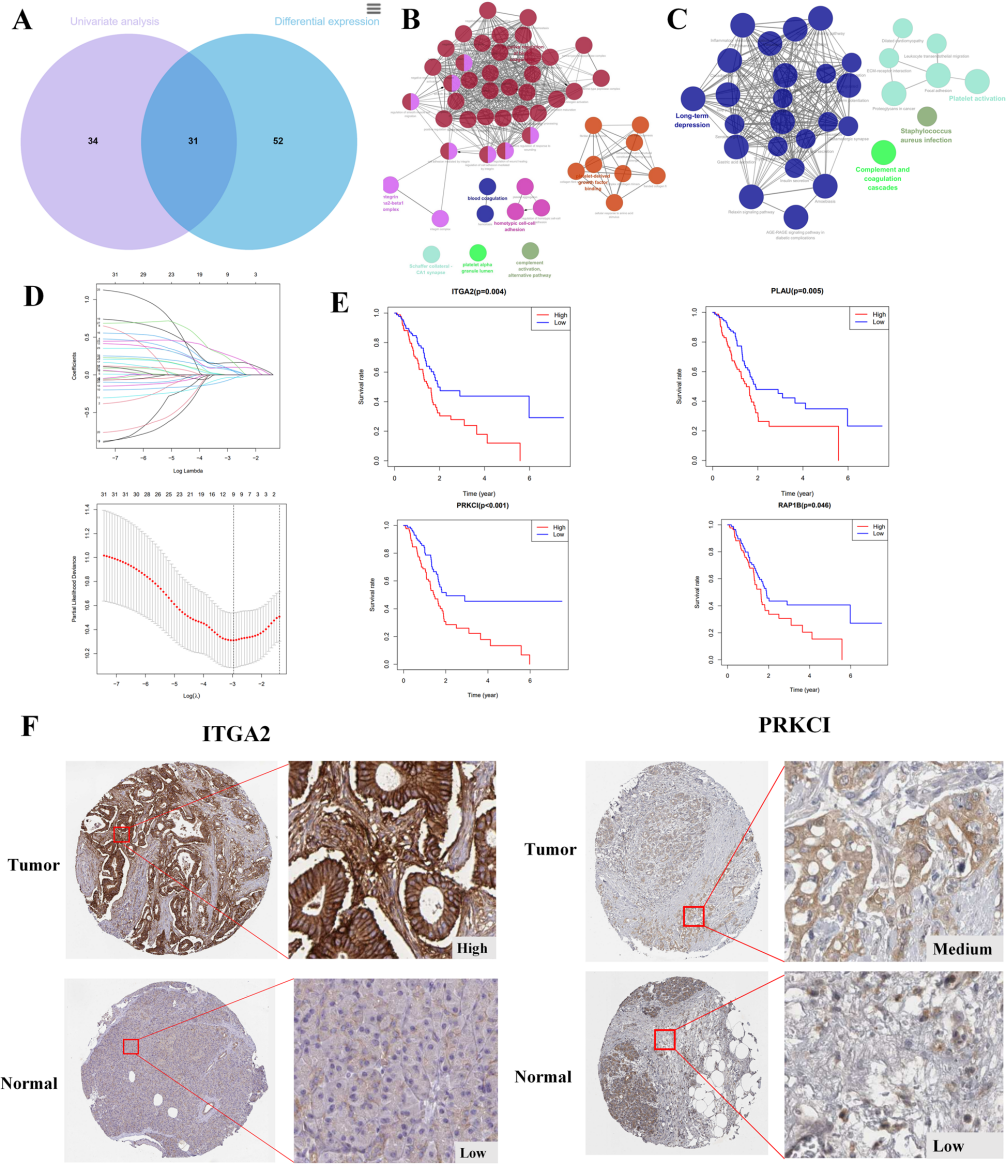
Enrichment analysis and screening out key genes. **A** Venn diagram between differential expression genes and genes associated with prognosis. The network of GO terms (**B**) and KEGG pathway (**C**) in ClueGO. **D** LASSO analysis of 31 genes. **E** The K-M curves of four risk genes. **F** Expression pattern of the 4 optimal PR-FRGs between tumor and normal specimens. **F** Immunohistochemistry staining of the 2 hub genes in tumor and normal tissues

A total of 65 and 83 genes were obtained through univariate analysis and differential analysis, respectively. Using intersection analysis, we identified 31 genes (Fig. 3A) and performed GO analysis (Supplementary Table 2) and KEGG analysis (Supplementary Table 3) using ClueGo to explore potential molecular function. GO analysis revealed that the hub genes were significantly enriched in the integrin alpha2-beta1 complex, homotypic cell–cell adhesion, blood coagulation and complement activation (Fig. 3B). The KEGG analysis indicated that these genes were mainly enriched in pathways, such as platelet activation, complement and coagulation cascades, long-term depression and proteoglycans in cancer (Fig. 3C). To identify the key genes among these 31 genes, we performed LASSO analysis with 1000-folds cross-validation and identified 9 hub genes, including PRKCI, RAP1B, PLAU, ITGA2, CFB, MYL12B, SERPINB2, GNAS and F5 (Fig. 3D). Supplementary Table 4 showed full names and functions of these hub genes. We further identified the top four genes (PRKCI, RAP1B, PLAU, and ITGA2) based on their value coefficient (Supplementary Table 5). K-M survival analysis revealed that all four genes were significantly associated with survival outcomes in PDAC (*p* < 0.05) (Fig. 3E). We also explored the protein expression level of PRKCI and ITGA2 in the Human Protein Atlas (HPA) database (Fig. 3F), and found that the expression level of both genes were significantly higher in tumor tissue than in normal tissues. Finally, we established a prognostic model based on the expression level and corresponding regression coefficients of each optimal PR-FRG. The formula for the risk score was: Risk score = Exp (PRKCI)*0.2606 + Exp(RAP1B)*0.2157 + Exp(PLAU)* 0.1600 + Exp(ITGA2)* 0.1007.

### Establishment of the prognostic model

**Fig. 4:**
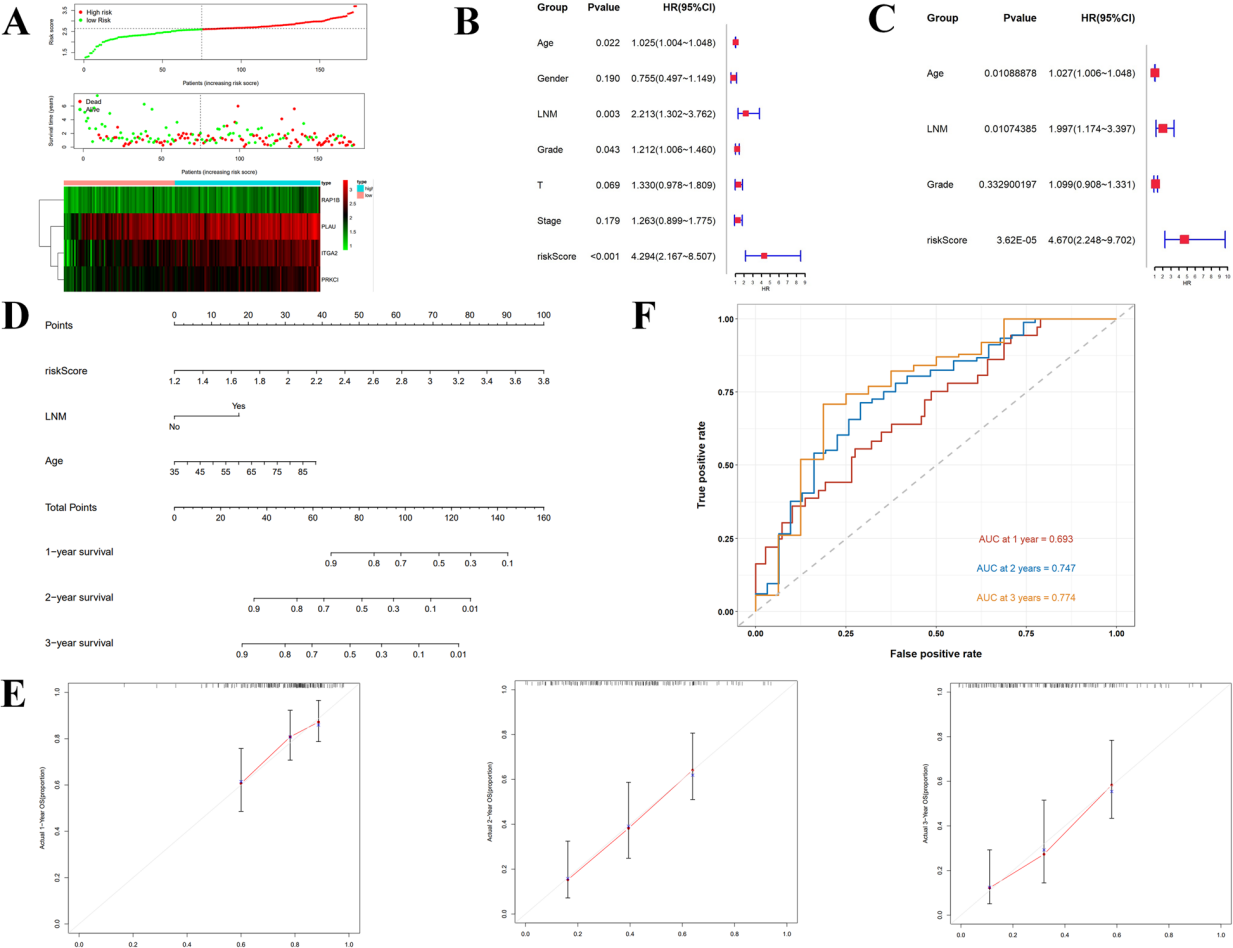
The construction of risk stratification model. **A** The curve of risk score, survival status and heatmap of the expression in four risk genes. Forrest plot of univariable Cox regression analysis (**B**) and multivariable Cox regression analysis (**C**) in PDAD. **D** Nomogram including age, Lymph node metastasis, risk score. **E** The 1-, 2- and 3-year calibration plots of the nomogram. **F** The 1-, 2- and 3-year ROC curves

In this study, we investigated the relationship between risk score and survival outcome of patients with PDAC. We stratified the patients into high- and low-risk groups based on the median risk score (2.639) and analyzed the survival status. As shown in Fig. 4A, PDAC patients in the high-risk group had a lower overall survival rate than those in the low-risk group. We further investigated the relationship between risk groups, coagulation clusters and survival status using a Sankey plot (Supplementary Fig. 2A). Our results indicated that the high-risk group had a poorer prognosis than the low-risk group, as confirmed by the K-M survival analysis (Supplementary Fig. 2B). Notably, this association remained significant across different clinical subgroups, including patients older than 65 years old, those with Lymph node metastasis, T3-4 stage and Grade1-2 tumors (Supplementary Fig. 2C-F). Additionally, using univariable and multivariate Cox regression analysis (Fig. 4B-C), we identified age, lymph node metastasis, and risk score as independent prognostic risk factors. To facilitate the clinical application of these findings, we constructed a nomogram based on the three identified prognostic factors (Fig. 4D) and validated its performance through 1-, 2-, and 3-years calibration curves (Fig. 4E) and a ROC curve analysis (Fig. 4F). Our results showed that the nomogram exhibited excellent performance in predicting survival outcomes in PDAC patients, with higher accuracy for 3-year prediction than for 1- and 2-year predictions. Overall, our findings suggest that the identified prognostic risk factors and the constructed nomogram may serve as valuable tools for personalized risk assessment and clinical decision-making in PDAC patients.

### Correlation with immune cells and drugs sensitiveness

**Fig. 5:**
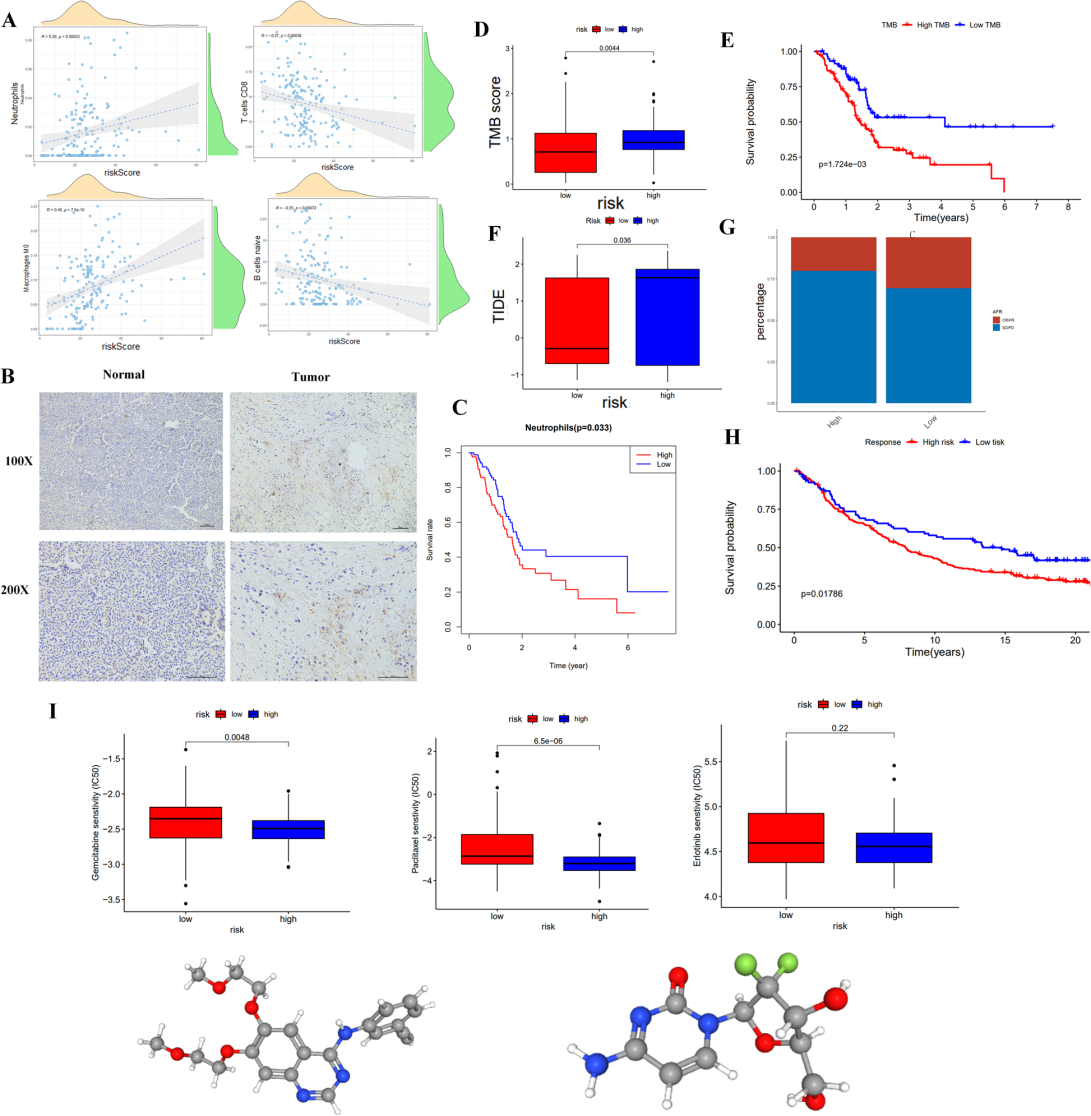
The analysis of correlation with immune cells and benefits of drug therapy. **A** The correlations between the risk score and immune cells, including Neutrophils, T cells CD8, B cells native and Macrophages M0. **B** Immunohistochemistry staining Neutrophils in normal and tumor tissues. **C** K-M survival curve of Neutrophils in PDAC. **D** The TMB analysis between high-risk and low-risk groups. **E** K-M curve of the high-TMB and low-TMB. **F** The TIDE score in two groups. **G** Bar graph illustrated the SD/PD and CR/PR in high-risk and low-risk groups. **H** K-M curve of high-risk and low-risk groups in IMvigor210. **I** The Drug sensibility of high-risk and low-risk groups for Gemcitabine, Paclitaxel and Erlotinib. **J** The 3D structure tomographs of the small-molecule drugs for Gemcitabine and Erlotinib

The study investigated the correlation between risk score and various immunocytes in PDAC. The results indicated a significant positive correlation between risk score and the infiltration of the neutrophils and macrophages M0, whereas T cells CD8 and B cells native showed an opposite correlation pattern (Fig. 5A). Given the crucial role of neutrophils in tumor immune microenvironment, the study further confirmed a higher level of Neutrophils infiltration in PDAC tissues than normal tissues using immunohistochemistry (Fig. 5B). Notably, the K-M survival analysis revealed that the patients with high neutrophil infiltration levels had a poor prognosis (*p* < 0.05) (Fig. 5C). Compared with low-risk group, high-risk group tended to high TMB scores compared to the low-risk group (*p* < 0.05) (Fig. 5D). Correlation analysis showed a positive association between TMB and risk score (Supplementary Fig. 3A). Additionally, the study demonstrated that PDAC patients with low TMB had a better prognosis than those with high TMB (Fig. 5E). These findings provide novel insights into the complex interplay between the tumor microenvironment and immune response in PDAC, and further investigations are warranted to uncover the underlying molecular mechanisms.

In addition, we have investigated the correlation between TIDE score and patient risk stratification, as well as the efficacy of anti-PD L1 immunotherapy in patients with different risk levels. Our findings revealed that the TIDE score was significantly higher in the high-risk group compared to the low-risk group (Fig. 5F). Furthermore, we utilized IMvigor210 to explore the effectiveness of immunotherapy and found that patients with CR or PR had better prognoses compared to those with PD or SD (Supplementary Fig. 3B). Notably, the high-risk group patients tended to exhibit higher proportion of SD/PD, while CR/PR were more frequently observed in the low-risk group (Fig. 5G). Additionally, the analysis of IMvigor210 revealed that patients in the low-risk group had better survival outcomes compared to those in the high-risk group (Fig. 5H). We also evaluated the response of patients to three common chemotherapeutic agents and observed that the low-risk group was more sensitive to traditional chemotherapeutic agents, including Gemcitabine and Paclitaxel. However, the high-risk group exhibited higher sensitivity to some targeted drugs, such as Erlotinib (Fig. 5I). The 3D structure tomography of Gemcitabine and Erlotinib, as explored by PubChem, is presented in Fig. 5J. These results suggest that TIDE score and patient risk stratification could be useful predictors for selecting appropriate treatment strategies and improving patient outcomes.

### Identify the hub gene ITGA2

**Fig. 6:**
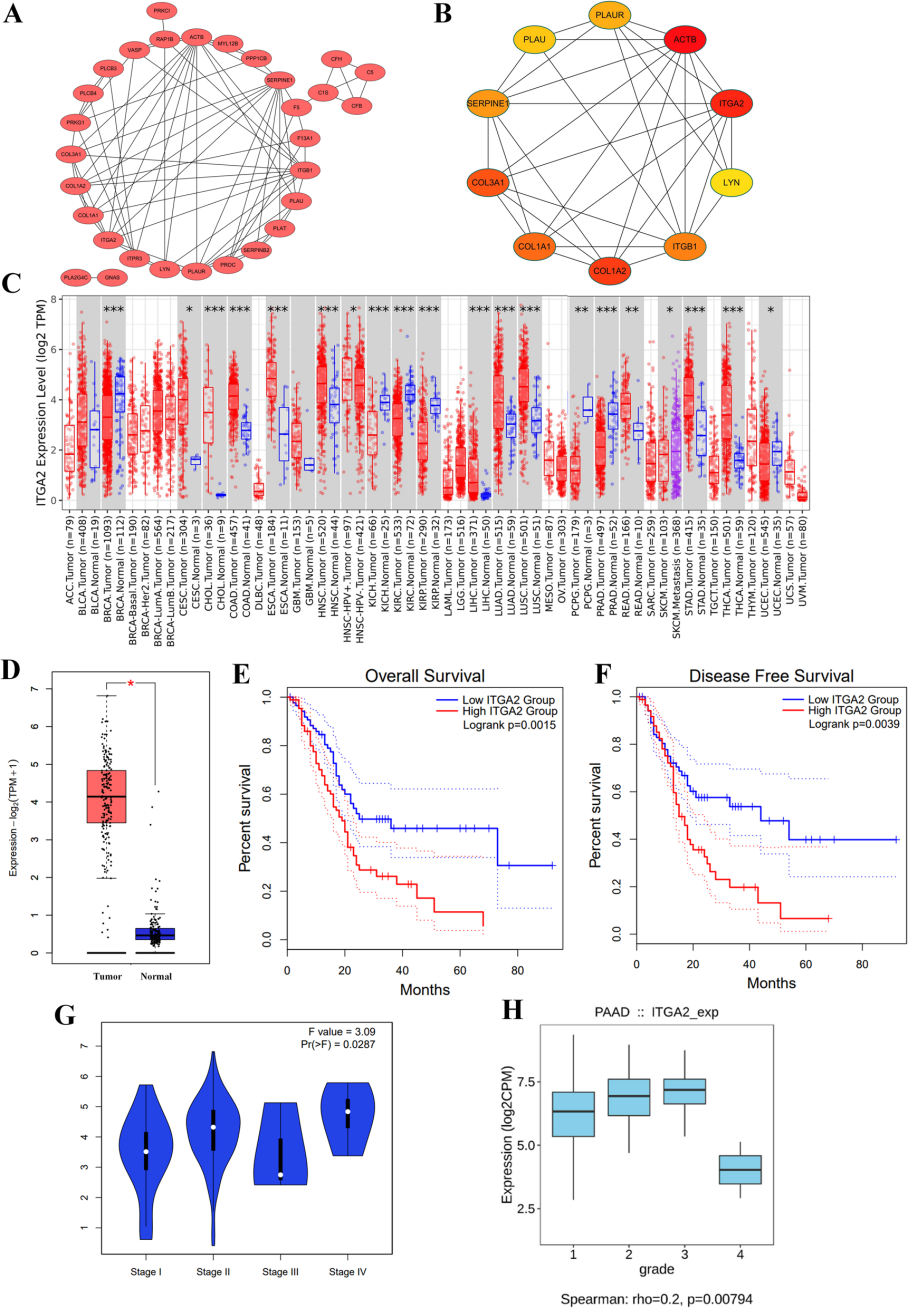
Identify the hub gene ITGA2. **A** 31 genes screened out by differential expression genes and genes associated with prognosis. **B** The top 10 genes by MCC algorithm in Cytoscape. **C** ITGA2 expression in pan-cancer. **D** ITGA2 was higher in TCGA-PDAC patients than that in GTEx control. **E** ITGA2 high expression had a poor survival outcome. **F** ITGA2 high expression had a short time in disease-free survival. It had the significant ITGA2 expression difference in tumor stage (**G**) and tumor grade **H**

The hub genes mentioned previously were visualized using Cytoscape, which enabled the acquisition of correlation values and node counts (Fig. 6A). The cytoHubba algorithm identified the top 10 genes, with red nodes highlighting their significance. Notably, ITGA2 and ACTB emerged as crucial genes, as depicted in Fig. 6B. After intersecting with four risk genes, ITGA2 was identified as the prime candidate, and its expression was analyzed in different tumors using TIMER2. Notably, the expression of ITGA2 was significantly higher in tumor tissues of CESC, CHOL, COAD, ESCA, HNSC, LIHC, LUAD, LUSC, READ, STAD and THCA than in normal tissues. However, the expression levels of ITGA2 were lower in BRCA, KICH, KIRC, KIRP, PCPG, PRAD and UCEC tumors than in normal tissues (Fig. 6C). As shown Fig. 6D, the expression of ITGA2 was higher in TCGA-PDAC patients than that in GTEx control. To assess the prognostic significance of ITGA2 in PDAC, we conducted survival analysis, and our results demonstrated that high ITGA2 expression was associated with poor prognosis and short DFS (Fig. 6E-F). We also investigated the correlation between ITGA2 expression and clinicopathological features and observed that the high ITGA2 expression was associated with late stage and high grade (*p* < 0.05) (Fig. 6G-H).

### Single cell sequencing analysis and immunohistochemical validation

**Fig. 7:**
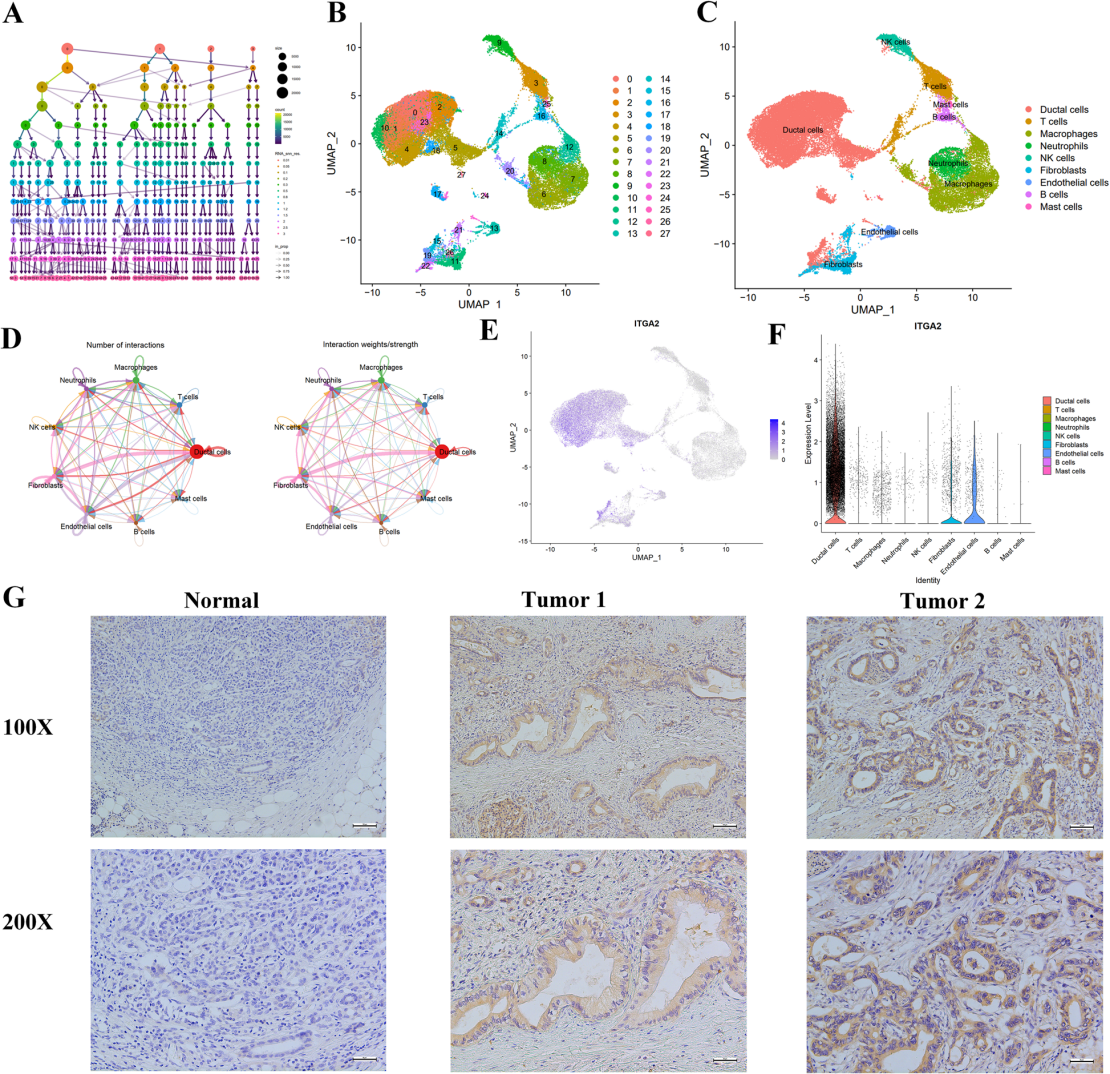
Single cell sequencing analysis and immunohistochemical analysis. **A** Set Resolution = 0.8 to obtain cell subgroups. **B** The UMAP map of all cells after quality control and standardization revealed 28 cell clusters marked with different colors. **C** 9 cell clusters according to gene marker annotated cell types. **D** An overview of cell–cell interactions. **E** The ITGA2 high expression cell clusters. **F** ITGA2 was expressed by ductal cells. **G** immunohistochemical staining for ITGA2

We analyzed single-cell RNA-seq data of 10 PDAC patients to resolve the architecture of the tumor microenvironment. We set Resolution = 0.8 (Fig. 7A) to obtain cell subgroups, and identified 28 clusters using uniform manifold approximation and projection (UMAP) based on all gene expression levels (Fig. 7B). Clusters of all cells were annotated as T cells (CD3E, CD3D, CD2), B cells (CD79A, CD19, MS4A1), Macrophages (CD163, CD68), Neutrophils (S100A8, CSF3R, S100A9), NK cells (FGFBP2, PRF1, GZMB), Mast cells (CPA3, TPSB2), Fibroblasts (COL1A1, ACTA2), Ductal cells (KRT19, EPCAM), and Endothelial cells (PECAM1, VWF) (Fig. 7C). CellChat was used to delineate intricate cell-to-cell communications and predict biologically significant findings from scRNA-seq data. Figure 7D showed the aggregated cell–cell communication network, suggesting the interaction strength and the cell types with significant changes, especially between ductal cells and fibroblasts. We further explored the expression profile of the ITGA2. We found that ITGA2 is primarily expressed in ductal cells, with a small amount of expression in endothelial cells and fibroblasts (Fig. 7E-F). Further research is needed to determine its intercellular function. In order to further verify the expression of the ITGA2 in PDAC tissue, we collected five normal pancreatic tissues (from donors who died of cardiovascular disease) and 15 pancreatic cancer patients, and performed immunohistochemistry. The results showed that the target gene was barely expressed in normal pancreatic tissue, but was significantly upregulated in pancreatic cancer. One sample of normal tissue and two samples of pancreatic cancer tissue were selected for immunohistochemical staining and are presented in Fig. 7G.

## Discussion

PC is a highly aggressive and lethal malignancies with poor prognosis, mainly due to the lack of sensitivity for Immunotherapy or chemotherapy and high recurrence post-operation [37]. Additionally, abnormal coagulation in patients with cancer leads to an increased risk of venous and arterial thromboembolism, which is a significant cause of death [38]. A study involving 1,015,598 tumor patients reported a venous thrombosis incidence of 3.4% and pulmonary embolism incidence of 1.1%, with PC having the highest incidence of venous thrombosis [39]. A recent report by Frere C showed that 152 PDAC patients (20.79%) had venous thromboembolism, with a median time of 4.49 months before its occurrence. Moreover, the study revealed that patients with venous thromboembolism had a poor prognosis and shorter disease-free survival [40]. Currently, PDAC is believed to be associated with various factors, such as systematic metabolism and gut microbiomes [41]. Hua Zhong conducted a large-scale MWAS analysis and discovered that five metabolites (alpha-glutamylglycine, glycylglycine, X-21735, X-24309 and X-21849) showed significant associations with PDAC risk. In addition, they identified that Flavonifractor sp90199495 might be involved in the metabolic network, which also potentially contributes to the risk of PDAC [42]. PDAC specific risk factors for thrombosis included abdominal surgery, neoadjuvant therapy, chemotherapy and central vein catheter [43]. Gemcitabine, a chemotherapy drug, had been linked to increased tissue factor activity and decreased tissue factor inhibitor activity, which could lead to thrombosis. Kim JS found that the incidence of venous thromboembolism was 10.6% in PDAC patients receiving gemcitabine chemotherapy and had a poor prognosis (*p* < 0.05) [44]. While deep vein thrombosis and pulmonary embolism are the most common types of venous thromboembolism in PDAC patients [45], there has been an increasing incidence of visceral thrombosis, which occurs in the hepatoportal venous system, including the portal, mesenteric and splenic vein. Mier-Hicks A reported that their study had an incidence of visceral thrombosis in PDAC patients as follows: portal vein (45%), mesenteric vein (26%), splenic vein (17%) [46].

It has been widely observed that malignant tumor and coagulation are closely linked [47]. A hypercoagulable state in PDAC arises due to elevated procoagulant factors and diminished anticoagulant factors, leading to an imbalance in the coagulation process. However, the underlying biological mechanisms responsible for this phenomenon remain elusive. it is perceived that tumor cells induce certain factors to maintain hypercoagulable state, such as tumor progression (KRAS and p53), procoagulant factors (tissue factor and PAI-1), mucin production and inflammatory factors. The activation of the coagulation cascade can facilitate tumor cell invasion and coagulation proteins also can also promote tumor migration and metastasis [48]. Mutations in oncogenes (e.g., TP53) can regulate several effectors of coagulation, and cancer driver genes (e.g., KRAS) may influence the risk of venous thromboembolism in various types of cancer [49]. Tissue factor, a receptor of FVII/FVIIa, activates the extrinsic coagulation progress. The TF-FVIIa complex can activate the PAR2 signaling pathway, leading to increase VEGF expression that promotes cancer progression. TF has been linked to venous thromboembolism and poor prognosis in PC patients [50]. Tumor can product multiple inflammatory factors, such as IL1, TNFα and VEGF, which can induce endothelial cell activation, promote leukocyte recruitment and platelet adhesion, and generate FVIII and TF that accelerate thrombosis [51]. However, little research has been conducted on the correlation between coagulation and tumor immune microenvironment in PDAC. Therefore, we aim to investigate the impact of coagulation-related genes on tumor progression, prognostic stratification, tumor immune microenvironment, and sensitivity to chemotherapy and immunotherapy.

A recent study has highlighted the significant role of the tumor immune microenvironment in the proliferation and metastasis of malignant tumors [52]. It is widely recognized that PD-1/PD-L1 plays a pivotal role in escaping immunological surveillance in PDAC. However, the immunosuppressive microenvironment in PDAC limits the therapeutic efficacy of PD-1/PD-L1 checkpoint inhibitors [53]. PD-1 immune checkpoint blockades using agents such as pembrolizumab and nivolumab has shown promise as an immunotherapeutic strategy melanoma and non-small-cell lung cancer [54]. In this study, we investigated the relationship between coagulation clusters and immune checkpoint. Our results demonstrated a significantly positive correlation between immune checkpoints (including PD-1 and PD-L1) and cluster 1, suggesting that PD-L1 inhibitors may be more beneficial for patients expressing cluster 1. Furthermore, our findings revealed significant differences in the abundance of various immune cells, including Neutrophil, Dendritic cells activated and Macrophage M0. Neutrophils can produce neutrophil extracellular traps (NETs) through histone citrullination, a post-translational modification catalyzed by peptidyl arginine deiminase-4 (PADI4) [55]. Studies have suggested that Neutrophil gelatinase-associated lipocalin is a prognostic biomarker for PDAC [56], while the CTSC-PR3-IL-1β axis can induce neutrophil reactive oxygen species production and formation of NETs, thereby promoting the metastatic growth of cancer cells in the lungs [57]. In our study, we validated the higher infiltration of neutrophil in PDAC patients compared to normal patients, emphasizing the important roles of neutrophil in PDAC.

The crucial role of ITGA2 in tumor progression and its correlation with clinical factors prompted us to conduct further investigations. Integrins, the heterodimeric transmembrane proteins composed of α and β subunits, are known to have significant involvement in various biological functions, such as inflammation, tumor progression and coagulation [58]. The α subunit is associated with the extracellular matrix, while the β subunit is correlated with the intracellular signaling cascades [59]. ITGA2, a glycoprotein of the integrin family, forms the heterodimer α2β1 with ITGB1, resulting in diverse biological functions. Studies have demonstrated the critical role of ITGA2 in tumor metastasis, invasion and angiogenesis [60]. Recently, a study highlighted that high expression of ITGA2 promotes ovarian cancer cell proliferation and resistance to albumin paclitaxel through the AKT/FOXO1 signaling axis [61]. Our analysis revealed that ITGA2 was overexpressed in various tumors and associated with poor overall survival and disease-free survival. Additionally, high expression of ITGA2 was correlated with late-stage tumors and high tumor grade.

Though this study identified two distinct cluster and investigated the correlation with tumor immune microenvironment, it is important to acknowledge the limitations that exist within the scope of the research. Firstly, the transcriptome data and clinical information obtained from public database, may not be entirely comprehensive, as incomplete follow-up information for some patients and a lack of standardization in tissues collection could impact the accuracy of the findings. Secondly, tumor heterogeneity was unavoidable due to the use of various platform and approaches in the data collection process, potentially influencing the conclusiveness of the study. Thirdly, in order to elucidate the biological mechanism and pathway in PDAC, it would be necessary to conduct additional in vitro and in vivo experiments.

## Conclusions

In our study, we demonstrated the correlation the coagulation-related genes with tumor immune microenvironment. The two types of patients stratified by our model differ in prognosis, immune infiltrate features and immunotherapy. The stratified model can predict the prognosis and calculate the potential benefits of drug therapy, thus providing valuable recommendations for personalized clinical treatment. Finally, we identified the hub gene ITGA2 and the expression of ITGA2 in PDAC had been confirmed by immunohistochemical analysis.

## Supplementary Information

**Additional file 1: Supplementary Figure 1.** (A) Heatmap of the coagulation-related genes between coagulation cluster and clinical factors. (B) Histogram of the proportion of different CNA types.

**Additional file 2: Supplementary Figure 2.** (A) Sankey plot revealed the correlation among the clusters, risk stratification and survival status. (B) K-M survival analysis of risk stratification model based on four genes. K-M survival analysis of risk stratification in different clinical subgroups including (C) older than 65 years old, (D) Lymph node metastasis, (E) T3-4 and (F) Grade1-2.

**Additional file 3: Supplementary Figure 3.** (A) The correlations between the risk score and TMB. (B) K-M curve of SD/PD and CR/PR group in IMvigor210.

**Additional file 4: Supplementary Table 1.** More details on 203 coagulation-related genes.** Supplementary Table 2.** the GO enrichment analysis of 31 hub genes.** Supplementary Table 3.** the KEGG pathways analysis of 31 hub genes.** Supplementary Table 4.** Full names and functions in the hub genes.** Supplementary Table 5.** the results of lasso analysis.

## Data Availability

The data used in this study have already been deposited in Gene Expression Omnibus (accession GSE205013) and TCGA database. The coagulation pathways under investigation were derived from the KEGG database (https://www.genome.jp/kegg/). The data bases referenced in the methods section of this article are all open access.
