# Supplementary figures and images for: The coagulation-related genes for prognosis and tumor microenvironment in pancreatic ductal adenocarcinoma
Source: BMC Cancer. 2023 Jun 29;23:601. doi: 10.1186/s12885-023-11032-9 (PMC10308640; doi:10.1186/s12885-023-11032-9)

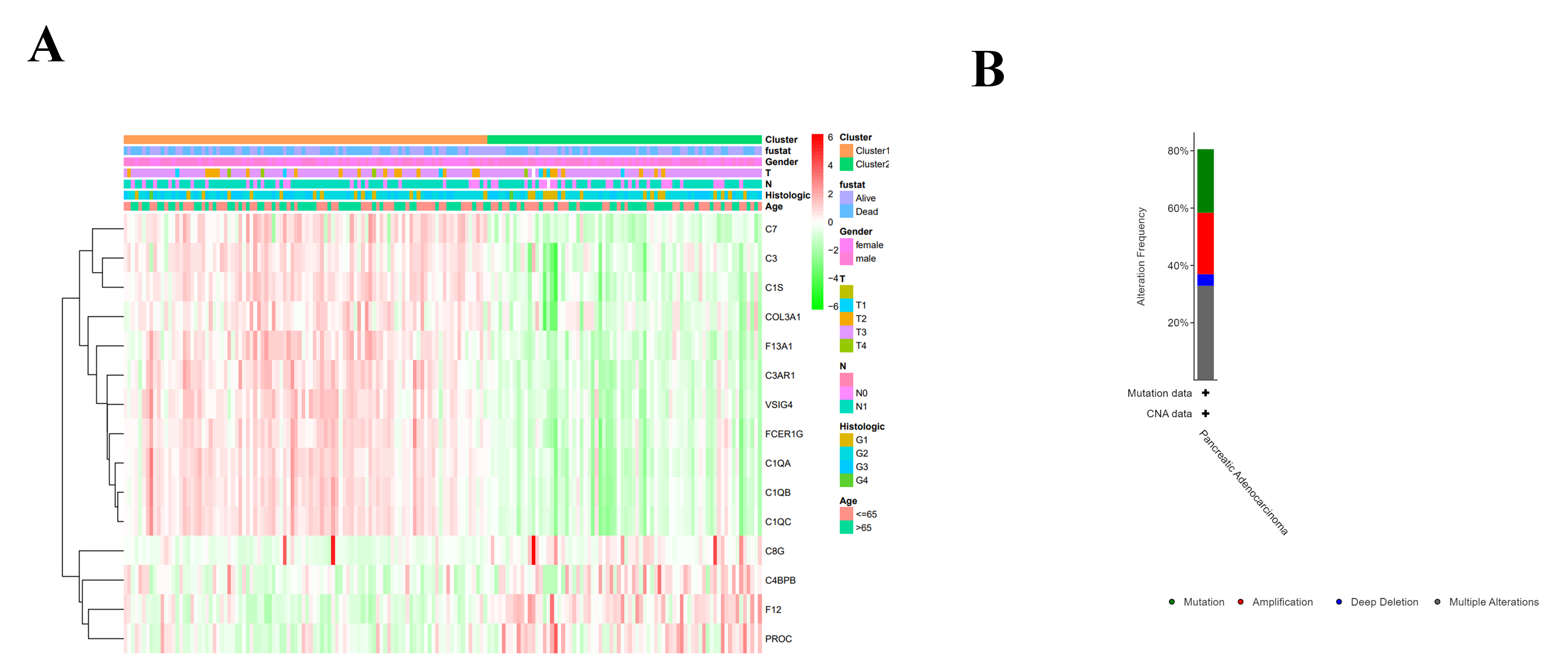

Supplement: Supplementary file 1 — Additional file 1: Supplementary Figure 1. (A) Heatmap of the coagulation-related genes between coagulation cluster and clinical factors. (B) Histogram of the proportion of different CNA types. [file 12885_2023_11032_MOESM1_ESM.tif]

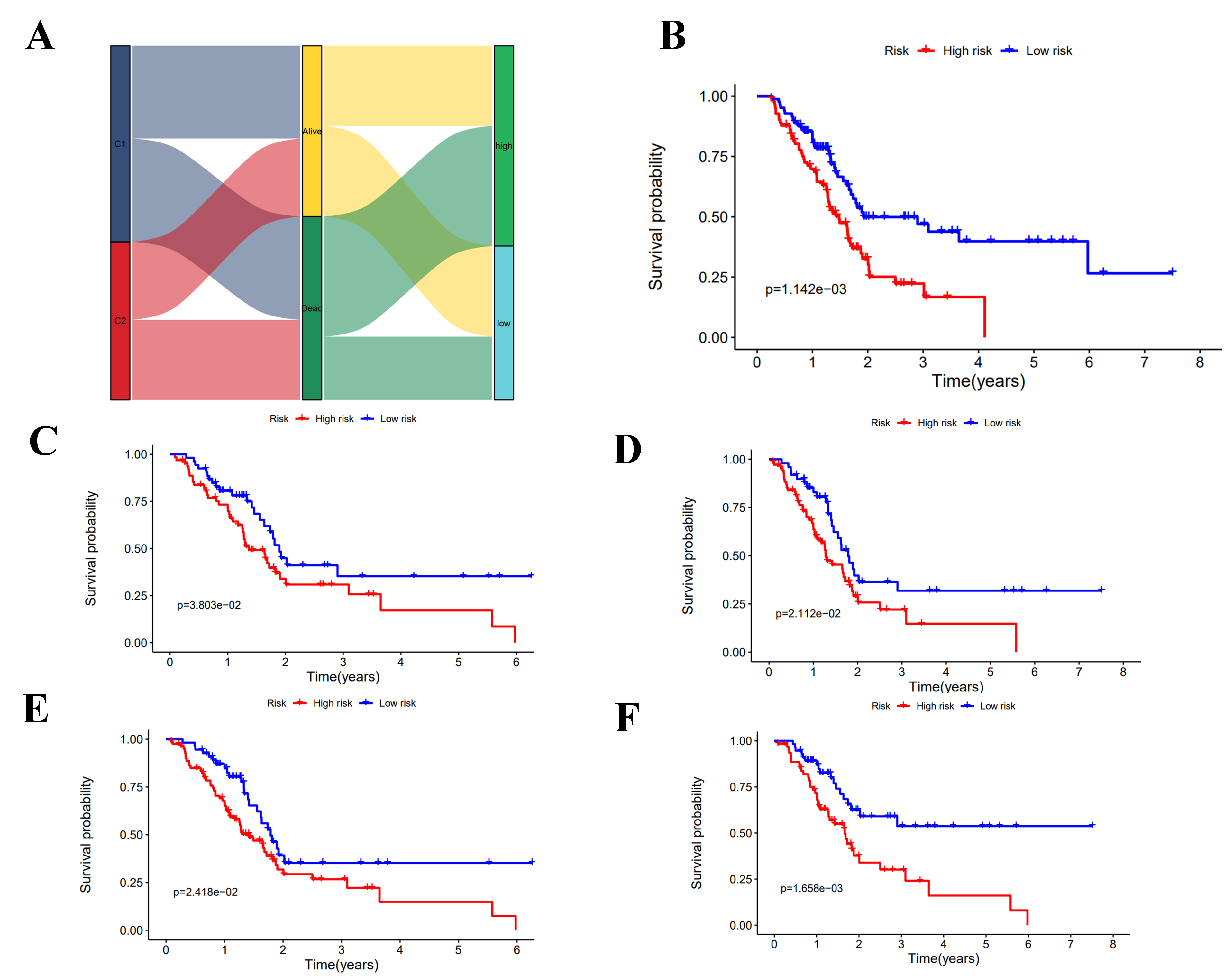

Supplement: Supplementary file 2 — Additional file 2: Supplementary Figure 2. (A) Sankey plot revealed the correlation among the clusters, risk stratification and survival status. (B) K-M survival analysis of risk stratification model based on four genes. K-M survival analysis of risk stratification in different clinical subgroups including (C) older than 65 years old, (D) Lymph node metastasis, (E) T3-4 and (F) Grade1-2. [file 12885_2023_11032_MOESM2_ESM.tif]

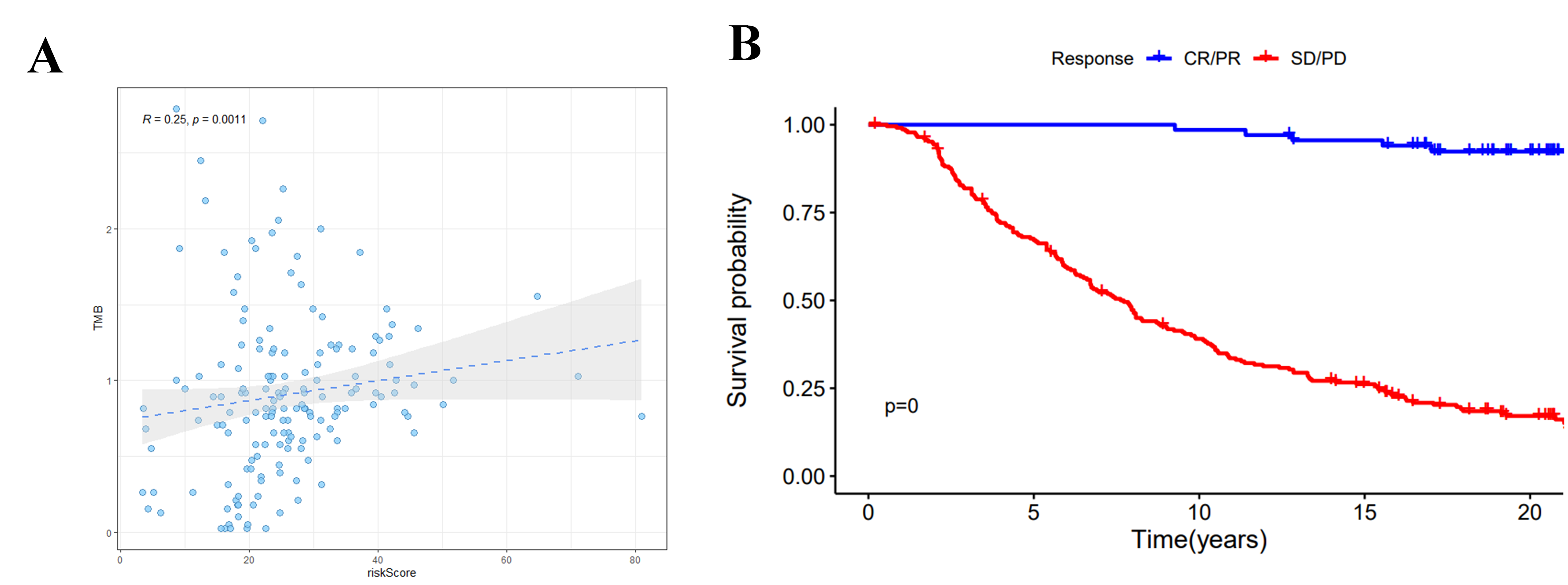

Supplement: Supplementary file 3 — Additional file 3: Supplementary Figure 3. (A) The correlations between the risk score and TMB. (B) K-M curve of SD/PD and CR/PR group in IMvigor210. [file 12885_2023_11032_MOESM3_ESM.tif]
