# Supplementary material for: The coagulation-related genes for prognosis and tumor microenvironment in pancreatic ductal adenocarcinoma
Source: BMC Cancer. 2023 Jun 29;23:601. doi: 10.1186/s12885-023-11032-9 (PMC10308640; doi:10.1186/s12885-023-11032-9)
Supplement: Supplementary file 4 — Additional file 4: Supplementary Table 1. More details on 203 coagulation-related genes. Supplementary Table 2. the GO enrichment analysis of 31 hub genes. Supplementary Table 3. the KEGG pathways analysis of 31 hub genes. Supplementary Table 4. Full names and functions in the hub genes. Supplementary Table 5. the results of lasso analysis. [file 12885_2023_11032_MOESM4_ESM.docx]

**Supplementary Figures**

Supplementary Table 1 More details on 203 coagulation-related genes.

| A2M | ACTB | ACTG1 | ADCY1 | ADCY2 | ADCY3 | ADCY4 | ADCY5 | ADCY6 | ADCY7 | ADCY8 |
| --- | --- | --- | --- | --- | --- | --- | --- | --- | --- | --- |
| ADCY8 | ADCY9 | AKT1 | AKT2 | AKT3 | APBB1IP | ARHGAP35 | ARHGEF1 | ARHGEF12 | BDKRB1 | BDKRB2 |
| BDKRB2 | BTK | C1QA | C1QB | C1QC | C1R | C1S | C2 | C3 | C3AR1 | C4A |
| C4A | C4B | C4BPA | C4BPB | C5 | C5AR1 | C6 | C7 | C8A | C8B | C8G |
| C8G | C9 | CD46 | CD55 | CD59 | CFB | CFD | CFH | CFHR1 | CFHR2 | CFHR3 |
| CFHR3 | CFHR4 | CFHR5 | CFI | CLU | COL1A1 | COL1A2 | COL3A1 | CPB2 | CR1 | CR1L |
| CR1L | CR2 | F10 | F11 | F12 | F13A1 | F13B | F2 | F2R | F2RL2 | F2RL3 |
| F2RL3 | F3 | F5 | F7 | F8 | F9 | FCER1G | FCGR2A | FERMT3 | FGA | FGB |
| FGB | FGG | FYN | GNA13 | GNAI1 | GNAI2 | GNAI3 | GNAQ | GNAS | GP1BA | GP1BB |
| GP1BB | GP5 | GP6 | GP9 | GUCY1A1 | GUCY1A2 | GUCY1B1 | ITGA2 | ITGA2B | ITGAM | ITGAX |
| ITGAX | ITGB1 | ITGB2 | ITGB3 | ITPR1 | ITPR2 | ITPR3 | JMJD7-PLA2G4B | KLKB1 | KNG1 | LCP2 |
| LCP2 | LYN | MAPK1 | MAPK11 | MAPK12 | MAPK13 | MAPK14 | MAPK3 | MASP1 | MASP2 | MBL2 |
| MBL2 | MYL12A | MYL12B | MYLK | MYLK2 | MYLK3 | MYLK4 | NOS3 | ORAI1 | P2RX1 | P2RY1 |
| P2RY1 | P2RY12 | PIK3CA | PIK3CB | PIK3CD | PIK3CG | PIK3R1 | PIK3R2 | PIK3R3 | PIK3R5 | PIK3R6 |
| PIK3R6 | PLA2G4A | PLA2G4B | PLA2G4C | PLA2G4D | PLA2G4E | PLA2G4F | PLAT | PLAU | PLAUR | PLCB1 |
| PLCB1 | PLCB2 | PLCB3 | PLCB4 | PLCG2 | PLG | PPP1CA | PPP1CB | PPP1CC | PPP1R12A | PRKACA |
| PRKACA | PRKACB | PRKACG | PRKCI | PRKCZ | PRKG1 | PRKG2 | PROC | PROCR | PROS1 | PTGIR |
| PTGIR | PTGS1 | RAP1A | RAP1B | RASGRP1 | RASGRP2 | RHOA | ROCK1 | ROCK2 | SERPINA1 | SERPINA5 |
| SERPINA5 | SERPINB2 | SERPINC1 | SERPIND1 | SERPINE1 | SERPINE2 | SERPINF2 | SERPING1 | SNAP23 | SRC | STIM1 |
| STIM1 | SYK | TBXA2R | TBXAS1 | TFPI | THBD | TLN1 | TLN2 | VAMP8 | VASP | VSIG4 |
| VSIG4 | VTN | VWF |  |  |  |  |  |  |  |  |

Supplementary Table 2 the GO enrichment analysis of 31 hub genes

| Ontology | Term | P value | Count |
| --- | --- | --- | --- |
| BP | blood coagulation | 4.07E-09 | 7 |
| BP | fibrinolysis | 3.21E-06 | 4 |
| BP | negative regulation of plasminogen activation | 6.48E-05 | 3 |
| BP | skin morphogenesis | 1.52E-04 | 3 |
| BP | negative regulation of fibrinolysis | 1.80E-04 | 3 |
| BP | complement activation, alternative pathway | 3.12E-04 | 3 |
| BP | proteolysis | 4.21E-04 | 6 |
| BP | cellular response to amino acid stimulus | 0.003769928 | 3 |
| BP | collagen fibril organization | 0.003896178 | 3 |
| BP | regulation of cell adhesion | 0.004154531 | 3 |
| CC | extracellular exosome | 1.05E-08 | 17 |
| CC | extracellular region | 5.49E-08 | 16 |
| CC | extracellular space | 1.41E-07 | 15 |
| CC | serine-type endopeptidase complex | 1.87E-06 | 4 |
| CC | integrin alpha2-beta1 complex | 6.16E-06 | 3 |
| CC | serine protease inhibitor complex | 2.05E-05 | 3 |
| CC | focal adhesion | 2.92E-05 | 7 |
| CC | glutamatergic synapse | 4.22E-05 | 7 |
| CC | blood microparticle | 5.62E-05 | 5 |
| CC | endoplasmic reticulum lumen | 7.39E-05 | 6 |
| MF | protease binding | 2.74E-05 | 5 |
| MF | protein binding | 8.51E-05 | 30 |
| MF | platelet-derived growth factor binding | 1.33E-04 | 3 |
| MF | serine-type endopeptidase activity | 2.34E-04 | 5 |
| MF | calcium ion binding | 0.001009 | 7 |
| MF | extracellular matrix structural constituent conferring tensile strength | 0.00192 | 3 |
| MF | integrin binding | 0.002078 | 4 |
| MF | receptor binding | 0.003406 | 5 |
| MF | collagen binding involved in cell-matrix adhesion | 0.007909 | 2 |
| MF | phospholipase C activity | 0.01888 | 2 |

Supplementary Table 3 the KEGG pathways analysis of 31 hub genes

| Id | Term | P value | Count |
| --- | --- | --- | --- |
| hsa04611 | Platelet activation | 3.49E-26 | 19 |
| hsa04610 | Complement and coagulation cascades | 4.08E-15 | 12 |
| hsa04730 | Long-term depression | 6.13E-08 | 7 |
| hsa04510 | Focal adhesion | 4.16E-07 | 9 |
| hsa05205 | Proteoglycans in cancer | 4.83E-07 | 9 |
| hsa04270 | Vascular smooth muscle contraction | 7.30E-06 | 7 |
| hsa04015 | Rap1 signaling pathway | 8.03E-06 | 8 |
| hsa04921 | Oxytocin signaling pathway | 1.62E-05 | 7 |
| hsa04750 | Inflammatory mediator regulation of TRP channels | 2.47E-05 | 6 |
| hsa04933 | AGE-RAGE signaling pathway in diabetic complications | 2.72E-05 | 6 |

Supplementary Table 4 Full names and functions in the hub genes

| N | Gene  symbol | Full name | Function |
| --- | --- | --- | --- |
| 1 | CFB | Complement Factor B | Upon activation of the alternative pathway, it is cleaved by complement factor D yielding the noncatalytic chain Ba and the catalytic subunit Bb |
| 2 | F5 | Coagulation Factor V | This factor circulates in plasma, and is converted to the active form by the release of the activation peptide by thrombin during coagulation. |
| 3 | GNAS | GNAS Complex Locus | Among its related pathways are ADORA2B mediated anti-inflammatory cytokines production and GPCR Pathway. |
| 4 | ITGA2 | Integrin Subunit Alpha 2 | Antibodies against this protein are found in several immune disorders, including neonatal alloimmune thrombocytopenia |
| 5 | MYL12B | Myosin Light Chain 12B | The activity of nonmuscle myosin II is regulated by phosphorylation of a regulatory light chain, such as MRLC2 |
| 6 | PLAU | Plasminogen Activator, Urokinase | Among its related pathways are ncRNAs involved in Wnt signaling in hepatocellular carcinoma and Innate Immune System |
| 7 | PRKCI | Protein Kinase C Iota | The PKC family comprises at least eight members, which are differentially expressed and are involved in a wide variety of cellular processes |
| 8 | RAP1B | RAP1B, Member Of RAS Oncogene Family | Members of this family regulate multiple cellular processes including cell adhesion and growth and differentiation |
| 9 | SERPINB2 | Serpin Family B Member 2 | Predicted to be involved in negative regulation of endopeptidase activity. |

Supplementary Table 5 the results of lasso analysis.

| Gene | Coef |
| --- | --- |
| PRKCI | 0.260556 |
| RAP1B | 0.215728 |
| PLAU | 0.160029 |
| ITGA2 | 0.100659 |
| CFB | 0.081543 |
| MYL12B | 0.078144 |
| SERPINB2 | 0.066232 |
| GNAS | -0.03545 |
| F5 | -0.06841 |

**Supplementary Figure**

Supplementary Figure 1 (A) Heatmap of the coagulation-related genes between coagulation cluster and clinical factors. (B) Histogram of the proportion of different CNA types.

Supplementary Figure 2 (A) Sankey plot revealed the correlation among the clusters, risk stratification and survival status. (B) K-M survival analysis of risk stratification model based on four genes. K-M survival analysis of risk stratification in different clinical subgroups including (C) older than 65 years old, (D) Lymph node metastasis, (E) T3-4 and (F) Grade1-2.

Supplementary Figure 3 (A) The correlations between the risk score and TMB. (B) K-M curve of SD/PD and CR/PR group in IMvigor210
